# A Human DUB Protein Array for Clarification of Linkage Specificity of Polyubiquitin Chain and Application to Evaluation of Its Inhibitors

**DOI:** 10.3390/biomedicines8060152

**Published:** 2020-06-04

**Authors:** Hirotaka Takahashi, Satoshi Yamanaka, Shohei Kuwada, Kana Higaki, Kohki Kido, Yusuke Sato, Shuya Fukai, Fuminori Tokunaga, Tatsuya Sawasaki

**Affiliations:** 1 Proteo-Science Center, Ehime University, Matsuyama 790-8577, Japan; yamanaka.satoshi.ze@ehime-u.ac.jp (S.Y.); s_k_0821@yahoo.co.jp (S.K.); e534062z@mails.cc.ehime-u.ac.jp (K.H.); f862001b@mails.cc.ehime-u.ac.jp (K.K.); 2 Center for Research on Green Sustainable Chemistry, Tottori University, Tottori 680-8552, Japan; yusato@tottori-u.ac.jp; 3 Department of Chemistry, Graduate School of Science, Kyoto University, Kyoto 606-8502, Japan; fukai@kuchem.kyoto-u.ac.jp; 4 Department of Pathobiochemistry, Graduate School of Medicine, Osaka City University, Osaka 545-8585, Japan; ftokunaga@med.osaka-cu.ac.jp

**Keywords:** deubiquitinating enzyme, polyubiquitin chain linkage, DUB inhibitor, wheat cell-free system, protein array technology

## Abstract

Protein ubiquitinations play pivotal roles in many cellular processes, including homeostasis, responses to various stimulations, and progression of diseases. Deubiquitinating enzymes (DUBs) remove ubiquitin molecules from ubiquitinated proteins and cleave the polyubiquitin chain, thus negatively regulating numerous ubiquitin-dependent processes. Dysfunctions of many DUBs reportedly cause various diseases; therefore, DUBs are considered as important drug targets, although the biochemical characteristics and cellular functions of many DUBs are still unclear. Here, we established a human DUB protein array to detect the activity and linkage specificity of almost all human DUBs. Using a wheat cell-free protein synthesis system, 88 full-length recombinant human DUB proteins were prepared and termed the DUB array. In vitro DUB assays were performed with all of these recombinant DUBs, using eight linkage types of diubiquitins as substrates. As a result, 80 DUBs in the array showed DUB activities, and their linkage specificities were determined. These 80 DUBs included many biochemically uncharacterized DUBs in the past. In addition, taking advantage of these active DUB proteins, we applied the DUB array to evaluate the selectivities of DUB inhibitors. We successfully developed a high-throughput and semi-quantitative DUB assay based on AlphaScreen technology, and a model study using two commercially available DUB inhibitors revealed individual selectivities to 29 DUBs, as previously reported. In conclusion, the DUB array established here is a powerful tool for biochemical analyses and drug discovery for human DUBs.

## 1. Introduction

Protein ubiquitination is a posttranslational protein modification involved in the regulation of numerous biological processes, such as cell growth, responses to many kinds of biotic and abiotic stresses, and pathogenesis. In the process of ubiquitination, ubiquitin, a small protein consisting of 76 amino acids, is covalently attached to lysine (K) residues in the target protein through its C-terminal glycine by three sequential steps catalyzed by three enzymes, the E1, E2, and E3 ligases [1]. Subsequently, additional ubiquitin is conjugated to the ubiquitin attached to the target protein or to the free ubiquitin through its seven lysine residues (K6, K11, K27, K29, K33, K48, and K63), or the initiation methionine (M1), for the formation of polyubiquitin chains. Each polyubiquitin chain linkage has distinct biological roles and creates the diversity in the functions of ubiquitin in cells, which is termed the ubiquitin code [2]. Therefore, the regulation of cellular polyubiquitin chains is a key issue for understanding their biological functions.

As a counteraction to polyubiquitin formation, deubiquitinating enzymes (DUBs) cleave the polyubiquitin chains or remove the ubiquitin molecules conjugated to target proteins, and therefore act as negative regulators of ubiquitin- and polyubiquitin-mediated cellular processes [3]. At least 98 DUBs are encoded in the human genome. Considering the fact that more than 600 E3 ligases are present in the human genome, multiple cellular functions of DUBs may overlap. Recently, dysfunctions of many DUBs have been reported to cause various kinds of diseases [4,5,6,7]. Especially, the excess expression of certain DUBs, such as USP7 and USP14, in cells leads to the protection of their target proteins from ubiquitination and subsequent proteasomal degradation, resulting in numerous cellular dysfunctions caused by the abnormal accumulation of these target proteins [8,9]. Therefore, DUBs are regarded as an important drug target.

Based on the structures of their catalytic domains, DUBs are divided into several families [3,10]. Previously, four families, ubiquitin specific protease (USP), ovarian tumor domain containing protease (OTU), ubiquitin C-terminal hydrolase (UCH), and Josephin, were thought to be cysteine protease DUBs with three catalytic core amino acid residues consisting of cysteine, histidine, and asparagine/aspartic acid [3]. More recently, however, two predicted cysteine proteases, motif interacting with Ub-containing novel DUB family (MINDY) and Zn-finger and UFSP domain protein (ZUFSP), were added to cysteine protease DUBs [11,12,13]. In contrast, the DUBs belonging to the JAB1/MPN/MOV34 metalloenzyme (JAMM) family are zinc-dependent metalloproteases [3,14].

Although eight types of polyubiquitin chain linkages exist in cells, the linkage specificity of the substrate polyubiquitin chain varies in individual DUBs. Some DUBs specifically cleave certain polyubiquitin chain linkages. For example, OTULIN and CYLD, known as negative regulators of NF-κB signaling, are highly specific to M1-linked polyubiquitin chain and both K63- and M1-linked polyubiquitin chains, respectively [15,16,17]. Cezanne, which is also a negative regulator of NF-κB, is the only DUB specific for K11-linked polyubiquitin chain [18,19]. In contrast, other DUBs, especially those belonging to the USP family, showed broad specificities for many polyubiquitin chains [20,21]. However, the linkage specificities of many DUBs have yet to be determined. In addition, the specificities of some DUBs are inconsistent in individual studies, probably due to differences in the protein expression system, the form of each recombinant DUB (e.g., full-length or only catalytic domain), and the assay to detect the activity of each DUB. Therefore, it would be optimal to prepare recombinant DUBs by the same expression system, and then determine their linkage specificities in a uniform assay platform.

Many DUBs have relatively large molecular weights with multiple functional domains, and it is difficult to produce the recombinant proteins in the full-length form. Indeed, previous biochemical analyses of the recombinant proteins often used artificially truncated recombinant proteins, with only the catalytic domain. However, the other domains, in addition to the minimum catalytic domain, are reportedly required for the activity and linkage specificity of some DUBs, such as USP13 and ATXN3 [22,23]. Therefore, the full-length intact form of each recombinant DUB would be important for its precise characterization. Here, we employed a wheat cell-free protein synthesis system (wheat cell-free system) for the preparation of recombinant full-length DUBs, because this expression system enables the synthesis of many kinds of eukaryotic proteins, including those with larger molecular weights [22,23]. Using this protein expression system, we established a human DUB protein array for in vitro analyses to reveal the activities and linkage specificities of most of human DUBs. In addition, using these active recombinant DUBs, we employed this array for evaluations of the selectivities of DUB inhibitors.

## 2. Materials and Methods

### 2.1. Materials

Eight linkage of diubiquitins were purchased from UbiQ Bio (Amsterdam, the Netherlands). PR-619 and SJB3-019A were purchased from LifeSensors (Malvern, PA, USA) and MedChemExpress (Monmouth Junction, NJ, USA), respectively. K48-, K63- and M1-linked tetraubiquitins were purchased from R&D systems (Minneapolis, MN, USA). The substrate ubiquitins, Mono-Ub, M1-Ub2, K48-Ub2, and M1-Ub4 were prepared as same procedure as previously described [24].

### 2.2. Construction of Expression Vectors Containing Human DUB cDNAs

The open reading frames (ORFs) of 72 DUB cDNAs were amplified by reverse transcriptase reactions, and subcloned into the pcDNA3.1 vector (Thermo Fisher Scientific, Waltham, MA, USA). Thirteen DUB cDNAs were obtained from the Mammalian Gene Collection (MGC). The ORF sequences of these DUBs were amplified by PCR, and then subcloned into the pEU vector (Cell Free Sciences, Yokohama, Japan) for the production of N-terminal AGIA-tag fusion proteins, using the In-Fusion system (Takara, Kusatsu, Japan).

### 2.3. Synthesis of Recombinant DUB Proteins

Using the pEU-AGIA-DUBs as in vitro transcription templates, the recombinant DUBs were synthesized with the wheat cell-free system (Cell Free Sciences), as previously described [25]. To confirm the synthesis of the DUB proteins, a two microliter portion of the crude translation mixture of each DUB was subjected to SDS-PAGE, followed by an immunoblot analysis using an anti-AGIA antibody.

### 2.4. Preparation of Anti-AGIA Antibody-Conjugated Magnetic Beads

The anti-AGIA antibody was prepared according to the procedure described previously [26]. For the preparation of magnetic beads conjugated with the anti-AGIA antibody, 50 µg of anti-AGIA antibody was attached to 50 µL of FG-NHS-beads, according to the manufacturer’s protocol (Tamagawa Seiki, Iida, Japan).

### 2.5. In Vitro Deubiquitination Assay

For the comprehensive DUB assay using the DUB protein array, a 10-microliter portion of the crude translation mixture of each DUB was mixed with 8 µL of the AGIA-magnetic beads, and incubated for 1 h at 4 °C with rotation. The beads were washed three times with the first wash buffer (20 mM Tris-HCl, pH 7.5, 500 mM NaCl, 0.05% Tween 20, 0.5% glycerol). The beads were additionally washed twice with the second wash buffer (20 mM Tris-HCl, pH 7.5, 150 mM NaCl, 0.05% Tween 20). The recombinant DUBs on the magnetic beads were then mixed with 10 µL portions of the substrate mixtures containing each of the eight diubiquitin linkages (final concentration of 2 µM), in 50 mM Tris-HCl, pH 7.5, 5 mM DTT, and the deubiquitination assay was performed for 3 h at 30 °C. The supernatant was separated from the recombinant DUBs on the beads by a magnetic stand, and mixed with SDS sample buffer. The diubiquitin and its cleaved product, monoubiquitin, in the supernatant were separated by SDS-PAGE. The proteins on the gel were stained with SYPRO Ruby Protein Gel Stain (Thermo Fisher Scientific). The stained proteins on the gel were detected with a TyphoonFLA imager (GE Healthcare, Chicago, IL, USA), using a 473-nm laser and a 580-nm emission filter. The obtained band intensities of monoubiquitin and diubiquitin in each gel were quantified by the ImageJ software. The activity of each DUB was calculated according to the following formula: Band intensity of monoubiquitin in presence of DUB/that of diubiquitin in absence of DUB × 100.

### 2.6. Inhibitor Assay Using AlphaScreen

The recombinant DUBs were captured with anti-AGIA tag-conjugated magnetic beads, using the same procedure as in the in vitro DUB assay. The recombinant DUB on the magnetic beads was combined with 10 µL of substrate mixture containing 2 µM of substrate ubiquitins shown in Figure S5, in 50 mM Tris-HCl, pH 7.5, 5 mM DTT, and the deubiquitination assay was performed for 3 h at 30 °C. The supernatant was separated from the recombinant DUB on the beads by a magnetic stand, and a 5 µL portion of each reaction was transferred to a 384-well OptiPlate (PerkinElmer, Waltham, MA, USA) containing AlphaScreen beads mix (100 mM Tris-HCl, pH 8.0, 100 mM NaCl, 1 mg/mL BSA, 0.1% Tween 20), 7.5 ng anti-DYKDDDDK (FUJIFILM Wako Pure Chemical Corporation, Osaka, Japan), 0.08 µL streptavidin-conjugated donor beads, and 0.08 µL protein A-coated acceptor beads (PerkinElmer), in a total volume of 25 µL. The OptiPlate was incubated for 1 h at room temperature, and the luminescent signal was detected by an EnVision plate reader (PerkinElmer).

## 3. Results

### 3.1. Preparation of Human DUB Protein Array

At first, 89 cDNAs encoding DUBs were subcloned into an expression vector specifically designed for the wheat cell-free protein synthesis, in the N-terminal AGIA-tagged form. The AGIA-tag is a high affinity-tag system composed of only 10 amino acids [26], and thus, this small tag is expected to minimally affect the native characters of the DUB proteins. The information about the DUBs used in this study is listed in Table S1. After the cell-free protein synthesis, the expression of the recombinant DUBs was confirmed by an immunoblot analysis using an anti-AGIA antibody (Figure S1). Among the 89 DUBs used for the cell-free protein synthesis, only one DUB, PRPF8, was not obtained, probably due to its large molecular weight (274 kDa). Finally, a DUB protein array (DUB array) containing 88 recombinant DUBs, including 54 USP, 16 OTU, four UCH, four Josephin, and 10 JAMM family members was prepared (Table 1, see the “Successfully synthesized” column).

### 3.2. Establishment of an In Vitro DUB Assay

**Figure 1:**
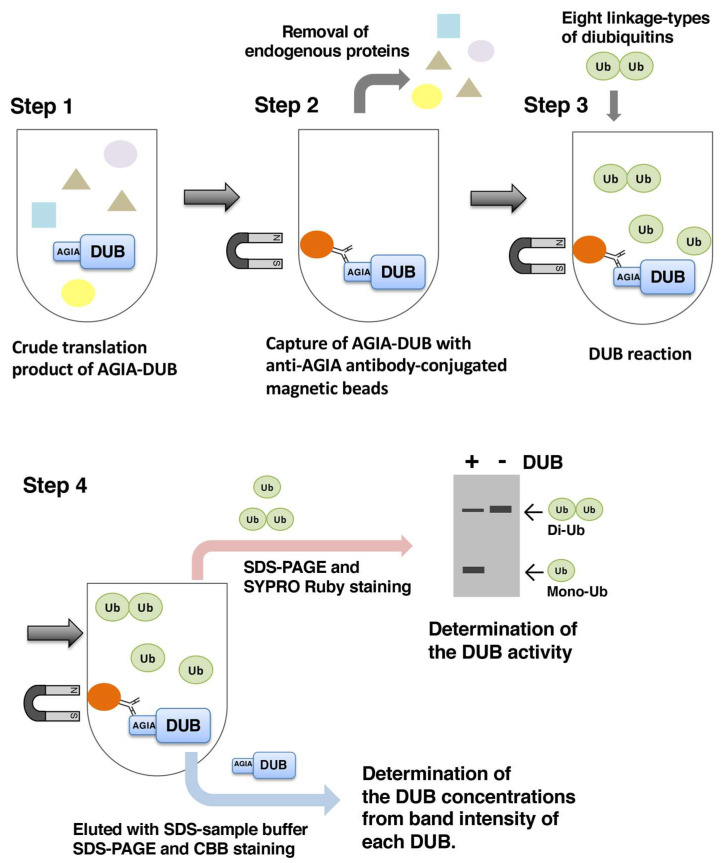
Overview of the in vitro DUB assay using eight linkage types of diubiquitin.

The DUB activities of these recombinant DUBs were evaluated. In this study, eight diubiquitin linkages (K6, K11, K27, K29, K33, K48, K63, and M1) were used as substrates, to clarify both the activity and linkage specificity of each DUB. Since the crude translation products of the cell-free system contained the distinct endogenous DUB activity from the wheat germ extract (Figure S2), the recombinant DUBs were purified to exclude the endogenous DUB activity. To skip time-consuming purification steps, such as His- and GST-purifications, we developed a simple on-bead cleavage assay that required only small amounts of recombinant DUBs, without contamination from endogenous DUBs. Due to its high affinity (Kd value: 4.9 × 10^−9^ M) and stable binding, the anti-AGIA antibody efficiently captured the AGIA-tagged proteins [26]. In order to improve the throughput in the purification step, we prepared anti-AGIA antibody-conjugated magnetic beads. After optimization, we established an in vitro DUB assay that required only 10 microliters of the crude cell-free translation mixture for each DUB reaction (Figure 1).

**Figure 2:**
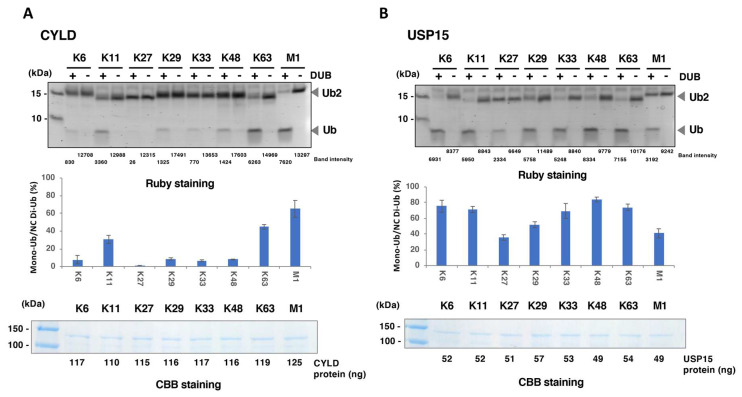
Representative results of on-beads cleavage assay. CYLD (**A**) and USP15 (**B**) were used as the linkage-specific DUB and the non-specific DUB, respectively. The diubiquitins and their cleaved product, monoubiquitin, were visualized with SYPRO Ruby staining (upper panel). The band intensities of di- and monoubiquitin in each reaction were quantified and the activity was calculated. mean ± S.D. (*n* = 3) (middle panel). Recombinant CYLD and USP15 were also visualized with Coomassie Brilliant Blue (CBB) staining (lower panel). Each amount of the recombinant protein was calculated from the band intensity and indicated below.

Two well-studied DUBs, CYLD and USP15, were used as the reference DUBs to evaluate the reliability of the assay. The fluorescent protein Venus was used as the negative control for these DUBs. The reaction products containing intact diubiquitin and its cleaved product, monoubiquitin, were separated by SDS-PAGE followed by staining with SYPRO Ruby, a high-sensitive protein stain with quantitative linearity. The DUB activity toward each diubiquitin was calculated by the ratio of cleaved monoubiquitin to the diubiquitin in the reaction. As shown in Figure 2A, CYLD mainly cleaved K63- and M1-diubiquitins, as previously demonstrated [15,17]. USP15 reportedly has broad linkage specificity [16], and our in vitro DUB assay revealed the cleavage of all eight diubiquitins (Figure 2B). In contrast, none of the diubiquitins were cleaved by the negative control protein Venus (Figure 2A,B, without DUB), indicating the negligible amount of endogenous DUB contamination from the wheat germ extract. In addition, the DUB activity obtained from three independent experiments were almost identical, indicating the good reproducibility of the assay (Figure 2A,B, middle panels). To further investigate the accuracy of the DUB assay, the amounts of recombinant DUB protein in the individual reactions were compared. The results confirmed that the amounts of the DUB proteins in the reactions with all eight linkages were almost identical (Figure 2A,B, lower panels). In addition, the concentrations of CYLD and USP15 were about 90 and 40 nM, respectively. Considering the previously reported biochemical study, these concentrations are sufficient for the detection of DUB activity. Taken together, we established a simple DUB assay to detect the cleavage activity of each DUB toward eight diubiquitin linkages.

### 3.3. Comprehensive DUB Assay Using the DUB Protein Array

**Figure 3:**
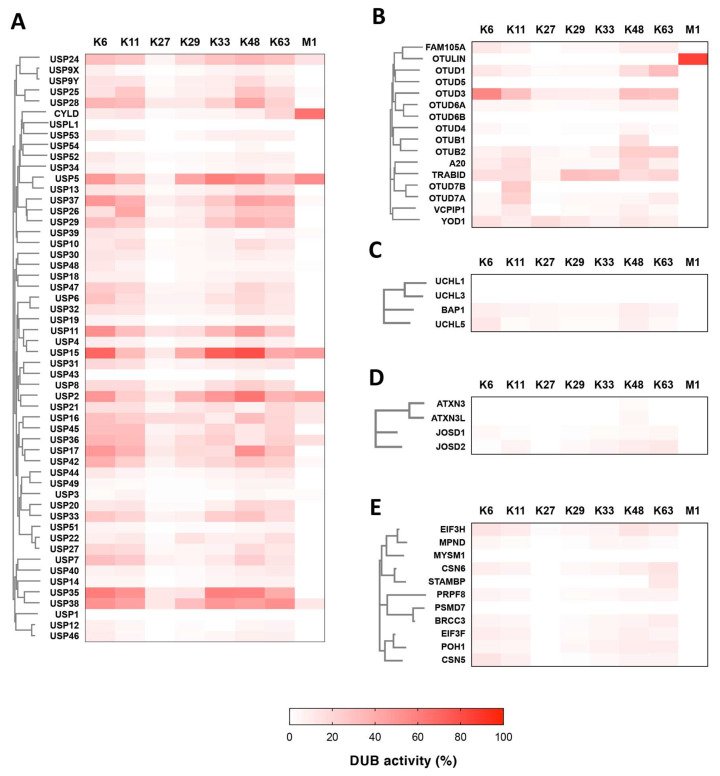
Determination of the linkage specificities of 89 DUBs. The assay results of the DUBs belonging to the USP (**A**), OTU (**B**), UCH (**C**), Josephin (**D**), and JAMM (**E**) families are indicated as a heatmap. The DUBs in each family were sorted, based on the phylogenetic tree.

Using the in vitro DUB assay shown in Figure 1 and Figure 2, we comprehensively investigated the activities and linkage specificities of all 88 DUBs in the protein array. The results of the SYPRO Ruby staining for the reactions of all 88 DUBs are shown in Figure S3. The activities of the DUBs were calculated by the same procedure as in Figure 2. The results of the DUB assays are clustered, based on the phylogenic tree constructed by the amino acid sequences of these DUBs, and are indicated as heat maps (Figure 3A–E). For each DUB reaction, the actual percentage of cleaved diubiquitin and the concentration of the DUB are listed in Table S2. Here, a DUB that cleaved at least one diubiquitin was defined as an “active DUB.” In this assay, 80 of the 88 DUBs showed DUB activity. In contrast, eight DUBs showed no activity. Notably, the DUBs used in this study included well-studied DUBs with unique linkage specificities to certain ubiquitin chains. For example, Cezanne and OTUB1, which are reportedly specific for K11- and K48- linked ubiquitin chains, respectively, showed the same linkage specificities as reported, indicating the accuracy of our assay. A summary of the assay is shown in Table 1 (see the “Active DUBs” column).

We then focused on the results of the DUB assays in individual DUB families. Regarding the USPs, 52 cleaved at least one diubiquitin (Figure 3A and Table 1). As previously reported [20,27], all USPs except for CYLD showed broad linkage specificities, and we did not find any novel USPs with strict linkage specificity. Interestingly, among these 52 active USPs, only nine USPs, USP2, USP5, USP15, USP16, USP21, USP24, USP36, USP38, and CYLD, showed distinct DUB activity toward M1-diubiquitin (more than 10% cleavage). In contrast to the USP family DUBs, some OTU family DUBs, such as OTULIN, OTUB1, and OTUD7B, showed strict linkage specificity toward one type of diubiquitin (Figure 3B), as previously reported [18,19]. Two OTUs, OTUD5 and OTUD6B, showed no activity, despite their sufficient concentrations in the assay (30 and 41 nM, respectively, Table S2). Surprisingly, FAM105A, which is considered as inactive DUBs [28], showed weak activity toward some diubiquitins. In the case of the UCH-family DUBs, only weak DUB activities toward seven K-linked diubiquitins, but not M1-diubiquitin, were observed for UCHL5 and BAP1, and no activity was detected with UCHL1 and UCHL3 (Figure 3C). For the Josephin family DUBs, JOSD1 and JOSD2 showed weak but distinct DUB activities toward several diubiquitin linkages (Figure 3D). ATXN3 and ATXN3L displayed faint activities toward K48-diubiquitin. These two DUBs reportedly cleave tetra- and longer ubiquitin chains [23,29], and our in vitro DUB assay revealed that they also cleave K48- and K63-tetraubiquitin chains, but not the M1-tetraubiquitin chain (Figure S4). As for the JAMM family, nine of the 11 DUBs had DUB activities. STAMBP showed strict specificity toward K63-diubiquitin and BRCC3 preferably cleaved K63-diubiquitin, as compared with other diubiquitin linkages (Figure 3E). The other active JAMMs exhibited broad specificities toward all seven K-linked diubiquitins, but not M1-diubiquitin.

### 3.4. Application of the DUB Array for the Evaluation of DUB Inhibitor Selectivity

**Figure 4:**
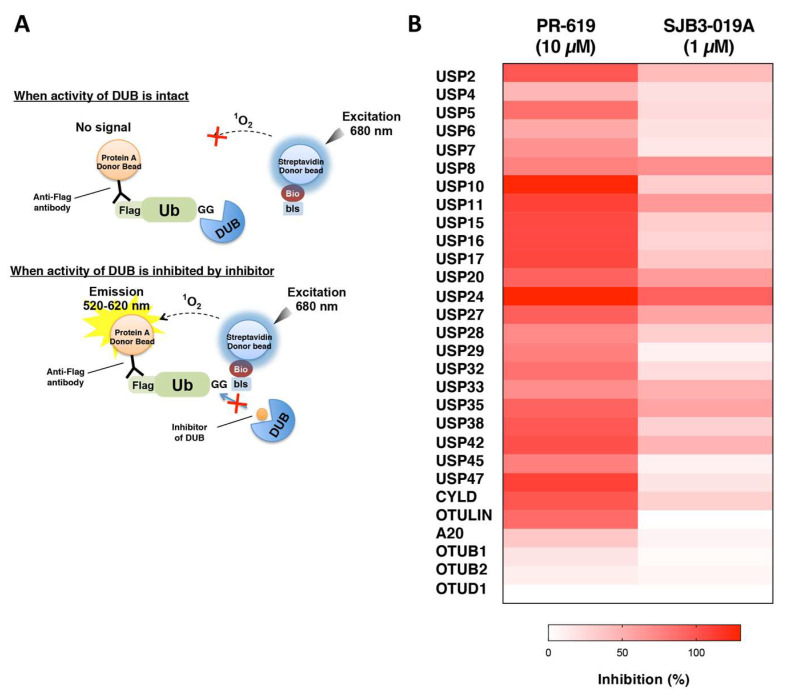
AlphaScreen-based evaluation of DUB inhibitors. (**A**) Schematic diagram of the assay. (**B**) The results of the evaluation of two DUB inhibitors, PR-619 and SJB3-019A. PR619 was used as a non-specific DUB inhibitor, and SJB3-019A was employed as relatively specific to some DUBs. The data are indicated as heatmaps.

Taking advantage of the large set of active DUBs, we applied the DUB array to develop an assay platform for evaluations of the selectivities of DUB inhibitors. Since the DUB assay using SYPRO Ruby staining was less quantitative and low throughput, we employed the AlphaScreen technology for measurements of the inhibitory effects of DUB inhibitors. This luminescent-based interaction assay is semi-quantitative and enables drug evaluations to be performed in a high-throughput manner with low-interference from various types of small chemical compounds [30,31,32]. The principle of the assay is shown in Figure 4A. To detect the activities of many kinds of DUBs with various linkage specificities, we designed four substrates based on mono-ubiquitin, K48- and M1-linked diubiquitins, and M1-linked tetraubiquitin for the AlphaScreen-based DUB assay (Figure S5). Using this assay, we successfully detected 29 DUBs. We next evaluated the inhibitory effects of two commercial compounds: PR-619, a non-specific DUB inhibitor, and SJB3-019A, which preferably inhibits some DUBs, such as USP1, USP2, and USP8, on these 29 DUBs. As expected, PR-619 strongly inhibited the activities of almost all DUBs tested, and SJB3-019A inhibited most of USPs but not OTUs, and its inhibitory efficiency was different for individual USPs (Figure 4B and Table S3). Interestingly, USP2 and USP8, which were both inhibited by SJB3-019A in a previous report, were also inhibited by 42% and 68%, respectively, in our evaluation. Based on these results, the DUB array established here is a useful tool for evaluations of DUB inhibitors.

## 4. Discussion

In this study, we successfully established a DUB array by the wheat cell-free system. As in the previous achievements [25,33], the expression system could synthesize 88 recombinant DUBs, including those with molecular weights greater than 100 kDa, without particular optimization for an individual DUB. The results of the in vitro DUB assay revealed that 80 of the 88 DUBs possessed activity toward at least one diubiquitin linkage. This means that more than 90% of the recombinant DUBs were synthesized as at least partially active forms; this is the first report of the activities and linkage specificities of such a large number of full-length DUBs. These results strongly indicate the robustness of the expression system, and the effectiveness of the on-bead DUB assay. In addition, because all of the full-length DUB proteins were synthesized and purified by identical methods, we could characterize these DUBs without the effects of differences in the experimental conditions, such as the protein expression system and the purification method. In this study, we aimed to know which linkage of diubiquitins each DUB can cleave. Therefore, the reaction time of the DUB assay is decided as 3 h, when most of DUB reaction is expected to be saturated. About the DUBs that showed weak activity; however, it is unclear whether these results were obtained by very slow but continuous enzymatic reaction of these DUBs, or by termination of the normal enzymatic reaction within the short period. Detailed time course experiments are needed to distinguish these two possibilities. Notably, the immunoblot analysis showed some DUBs such as USP26 and USP42 were synthesized as multiple molecular weight probably caused by unexpectable termination of peptide synthesis during in vitro translation (Figure S1). Although in vitro DUB assay detected the activity of these DUBs in Figure 3A, further validation is required for these DUBs to evaluate the activity and linkage specificity using full-length proteins without truncation. Focusing on the eight inactive DUBs, USPL1 was reported as an isopeptidase for SUMO, but not for ubiquitin [34], and OTUB6 was described as an inactive DUB in two previous reports [18,21]. Therefore, it is highly possible that these two DUBs are inactive toward the polyubiquitin chain. Among the other inactive DUBs, USP1 reportedly requires a binding partner protein UAF1, for activation [35]. However, other study showed that USP1 in the absence of UAF1 still possessed distinct activity [20]. Further analysis to check the activity of USP1 in presence or absence of UAF1 is thought to be required to clarify whether USP1 synthesized here is in a potentially active or completely inactive form because of improper folding. In addition, since the activation of OTUD5 requires its phosphorylation [18], it is reasonable that unphosphorylated form of OTUD5 in this study showed no activity. In a future study, the DUB assay should be performed in presence of these additional activation factors such as addition of binding partner protein and post-translational modifications.

Comparing our data with the results from other biochemical studies, the linkage specificities of many DUBs detected here are similar to the previously reported specificities (see Table S2). Especially, in the case of the well-studied OTU-family DUBs, our data were approximately consistent with previous reports [18,21]. The only exception is that the FAM105A has weak but distinct activity in this study (Figure 3B). This is surprising for us because FAM105A lacks catalytic core cysteine residue and was reportedly inactive DUB [28]. Further analysis is necessary to validate our result using mutant FAM105A proteins with substitutions of the amino acid residues such as those corresponding to the position of catalytic core (Asp139, His350, and His352). Similarly, the linkage specificities of the USP-family DUBs in our study were comparable with previous data [20,21], although some differences existent between our data and the previous data (e.g., capability to cleave M1-diubiquitin in USP4 and USP6). Although the USP family DUBs showed broad specificity toward many diubiquitin linkages, as previously reported, only nine USPs cleaved M1-diubiquitin (Figure 3A). The fact that USPs such as USP11, 17, 35, and 37 significantly cleave the isopeptide bonds of seven diubiquitins, but failed to cleave M1-diubiquitin, strongly suggests the requirement of specific structures in addition to the basal USP catalytic domain for the recognition and cleavage of the peptide-bond of the M1-ubiquitin chain by USPs.

For other DUB families, we detected the weak but distinct activities of UCHL5 and BAP1, although neither our study nor those by other groups detected the activities of UCHL1 and UCHL3. We also detected the activities of Josephin family DUBs, JOSD1 and JOSD2, by the DUB assay using diubiquitin (Figure 3D), and those of ATXN3 and ATXN3L using tetraubiquitins as the substrate (Figure S4). The JAMM-family DUBs in our study also have DUB activities, except for MYSM1 and PSMD7. Consistent with previous reports [21,36], STAMBP showed high linkage specificity to K63-diubiquitin in our assay. Many of the other JAMM-family DUBs showed broad linkage specificities, but all of the active JAMM-family DUBs failed to cleave M1-diubiquitin. Through the comprehensive DUB assay, we confirmed that only some USP-family DUBs and OTULIN have the ability to cleave M1-diubiquitin, indicating the peculiarity of peptide bond cleavage between ubiquitins, but not iso-peptide bond cleavage by many DUBs. Taken together, our study revealed the linkage specificities of many biochemically uncharacterized DUBs, and these results provide valuable information for many researchers engaged in DUB studies. However, in comparison with the USP- and OTU-family DUBs, the DUBs belonging to the other three families showed relatively weak activities. Even among the USP- and OTU-family DUBs, a few DUBs showed relatively weak or almost no activities (Figure 3). This might be caused by the particular characteristics of these families or alternatively, our assay was performed under inappropriate conditions, such as the length of ubiquitin chain, the buffer composition, or the absence of an interaction partner. As for the substrate ubiquitin, we used the diubiquitins in this study, since all eight linkage types of diubiquitins but not of longer ubiquitin chains are commercially available now. However, some DUBs such as ATXN3 and ATXN3L prefer longer ubiquitin chain than diubiquitin. In addition, longer ubiquitin chain than diubiquitin are reported to be exist in cells; thus, longer ubiquitin chains are thought to be more physiological substrate for many DUBs. Furthermore, some DUBs required interaction partner for their activation, besides USP1 and its interaction partner UAF1 described above. For example, the activity of UCHL5 was significantly enhanced by binding with its interactor, Adrm1 [37]. Because purified recombinant DUBs were used in the DUB assay demonstrated here, it is possible that we overlooked the potential activities of these DUBs, due to the lack of the interaction partners. To know the enzymatic character of the DUBs accurately, further analysis to identify the unknown interaction partners for DUBs are needed.

Taking advantage of the many active DUBs, we utilized the DUB array as an evaluation panel for the selectivities of DUB inhibitors. As described in the introduction, many DUBs are considered as attractive drug discovery targets, and indeed, an increasing number of reports have described the development of specific inhibitors for target DUBs [8,9,38,39,40]. However, Ritorto’s excellent work revealed that many of these DUB inhibitors, including one developed as a specific inhibitor for the target DUB, showed broad selectivities toward numerous DUBs from different families [21]. This is mainly caused by the structural similarity of the catalytic domains of DUBs. Especially, the structure of the catalytic triad and the mechanisms of peptide- and isopeptide-bond hydrolysis by nucleophilic attack are conserved in almost all cysteine protease DUBs, even from different families [21,41]. Therefore, the development of chemical compounds that specifically inhibit only the targeted DUBs is considered to be a challenging issue. Recently, specific DUB inhibitors for USP7 have been reported by two research groups [42,43]. In both studies, the selectivities of the lead compounds and their derivatives were biochemically evaluated using about 40 recombinant DUBs, and highly specific inhibitors for USP7 were eventually obtained by structural expansion of the lead compound. These achievements strongly indicate the importance of accurate biochemical evaluations using many recombinant DUBs. In this study, we established an assay platform to evaluate the selectivities of DUB inhibitors, using the AlphaScreen system. In a model study using two commercial DUB inhibitors, we properly evaluated the selectivity of each DUB inhibitor (Figure 4B), indicating the good potential of the assay. However, only 29 DUBs from the USP- and OTU-families were available for this evaluation. The major reason for this is thought to be the limitation of the substrate ubiquitins, since only monoubiquitin, K48-diubiquitin, M1-diubiquitin, and M1-tetraubiquitin were used. In a future study, we will prepare additional substrate ubiquitins, such as K63-diubiquitin, for the AlphaScreen to detect the activities of K63-specific and -preferring DUBs.

In conclusion, the DUB protein array provided information about the linkage specificities of many human DUBs, which is valuable for many researchers engaged in DUB analyses. The array is also expected to facilitate the development of DUB inhibitors in the future.

## Figures and Tables

**Table 1:** Summary of the expression of recombinant deubiquitinating enzymes (DUBs) and the in vitro DUB assay. USP: ubiquitin specific protease; OTU: ovarian tumor domain containing protease; UCH: Ubiquitin C-terminal hydrolase; JAMM: JAB1/MPN/Mov34 metalloenzyme.

Family (in Human Genome)	Expression Construct	Successfully Synthesized	Active DUBs	Inactive DUBs
USP (56)	54	54	52	2
OTU (17)	16	16	14	2
UCH (4)	4	4	2	2
Josephin (4)	4	4	4	0
JAMM (12)	11	10	8	2
Total (93)	89	88	80	8

## Supplementary Materials

The following are available online at https://www.mdpi.com/2227-9059/8/6/152/s1. Figure S1. Expression of the human DUBs; Figure S2. The wheat germ extract has distinct DUB activity; Figure S3. The result of in vitro DUB assay; Figure S4. The result of DUB assay of ATXN3 and ATXN3L using tetraubiquitins as substrate; Figure S5. The schematic diagram of substrate ubiquitins for AlphaScreen-based DUB assay; Table S1. The list of all human DUBs; Table S2. The results of DUB assay using diubiquitins; Table S3. The results of the AlphaScreen-based evaluation of DUB inhibitors in Figure 4B.

## Abbreviations

DUB	Deubiquitinating enzyme
USP	Ubiquitin specific protease
OTU	Ovarian tumor
UCH	Ubiquitin C-terminal hydrolase
JAMM	JAB1/MPN/Mov34
CBB	Coomassie Brilliant Blue
