# Supplementary material for: A Human DUB Protein Array for Clarification of Linkage Specificity of Polyubiquitin Chain and Application to Evaluation of Its Inhibitors
Source: Biomedicines. 2020 Jun 4;8(6):152. doi: 10.3390/biomedicines8060152 (PMC7344921; doi:10.3390/biomedicines8060152)
Supplement: Supplementary file 1 [file biomedicines-08-00152-s001.zip › Supplemental materials/Supplemantary Figures and the caption for Supplemantary Tables.pdf]

Figure S1

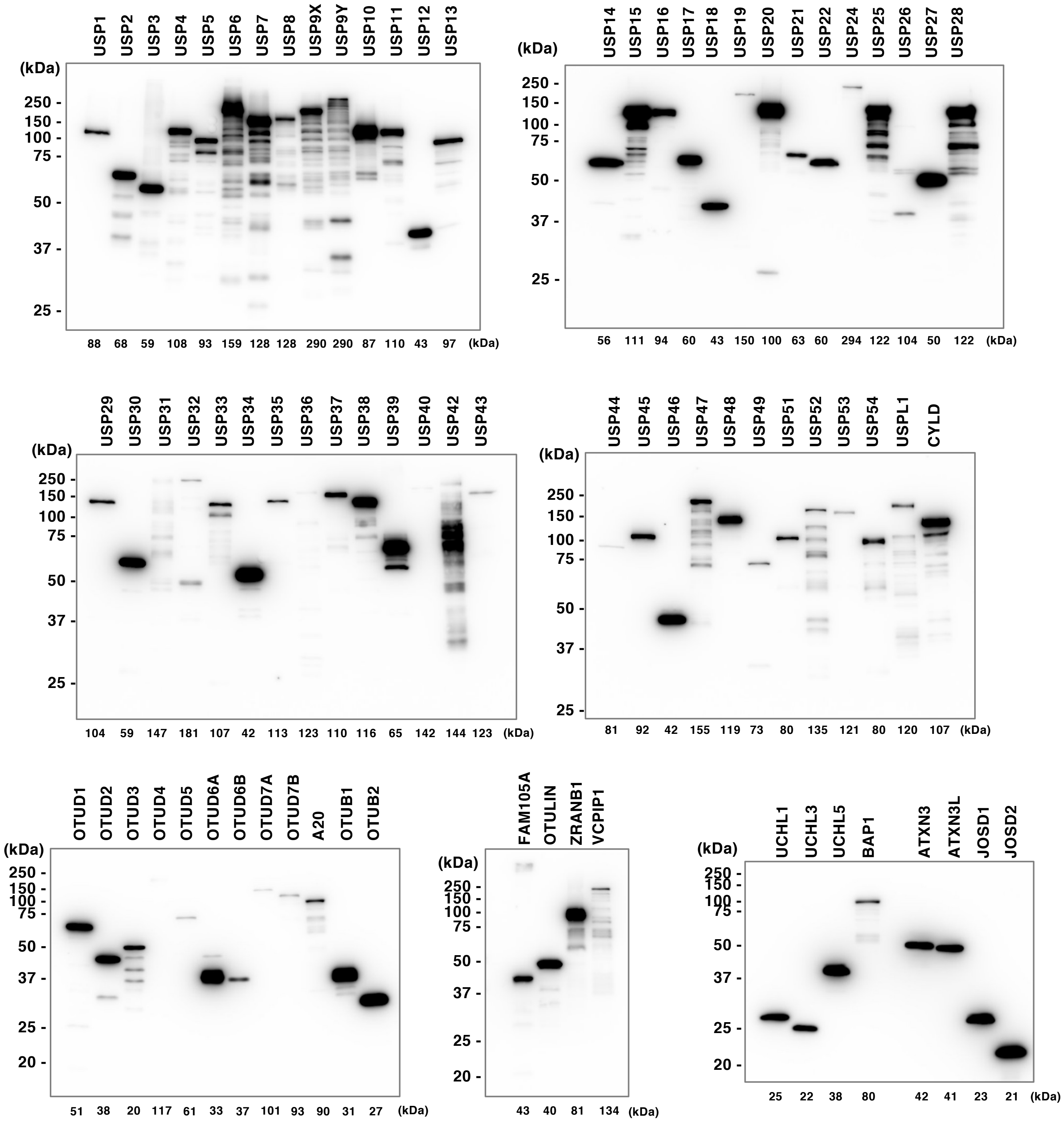

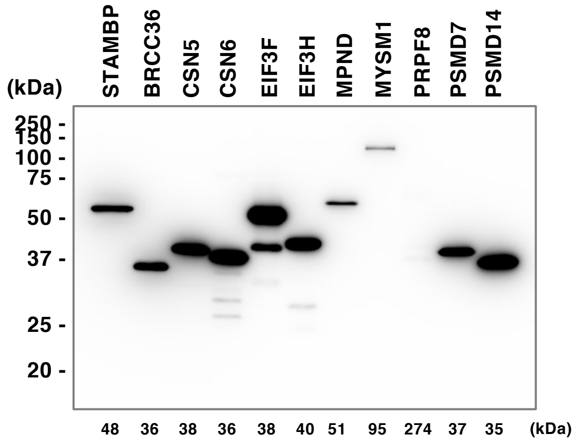

Figure S1. Expression of the human DUBs. The 89 recombinant DUBs were synthesized by the wheat cell-free system, and subjected to SDS-PAGE followed by immunoblot analysis using anti-AGIA antibody.

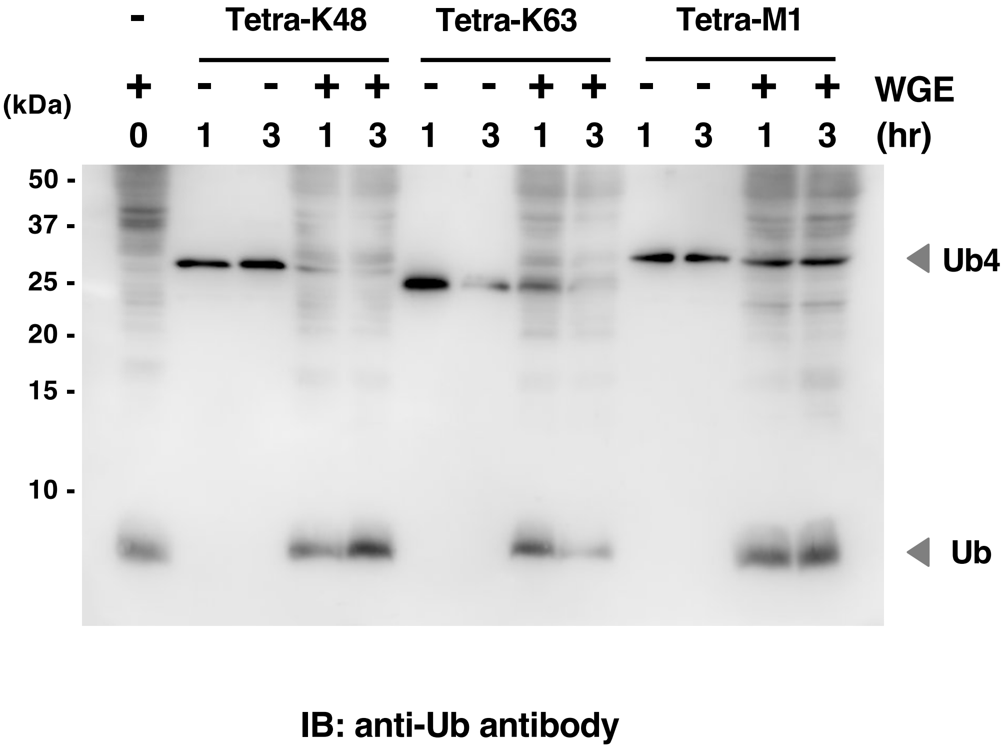

Figure S2. The wheat germ extract has distinct DUB activity. K48-, K63-, and M1-tetraubiquitins were incubated with 0.75 μL of the wheat germ extract (WGE) for indicated time. The reaction products were separated by SDS-PAGE followed by immunoblot analysis using anti-ubiquitin antibody.

Figure S3

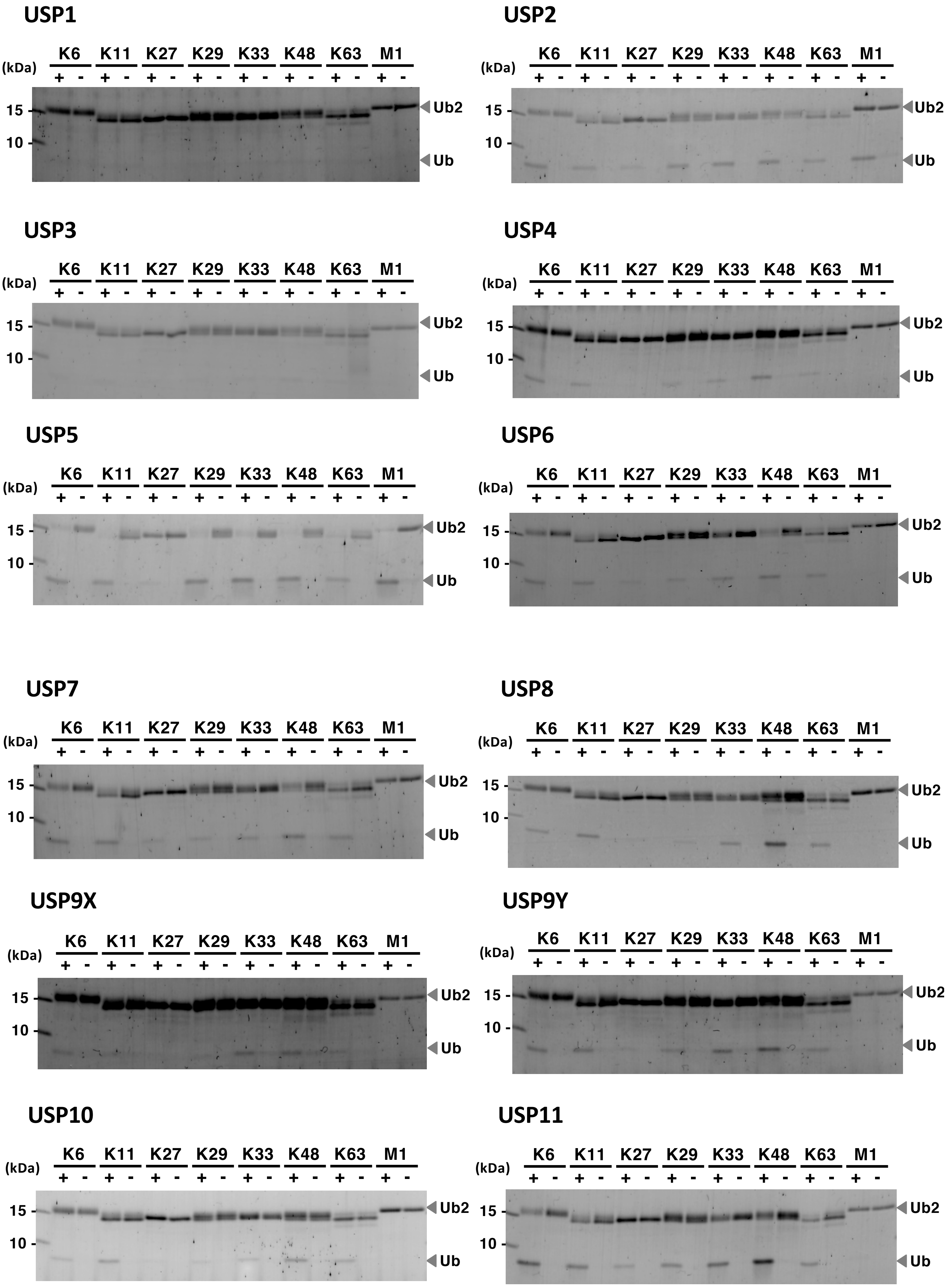
USP1
USP2
USP3
USP4
USP5
USP6
USP7
USP8
USP9X
USP9Y
USP10
USP11
K6 K11 K27 K29 K33 K48 K63 M1
(kDa)
+ - + - + - + - + - + - + - + -
15
10
Ub2
Ub

Figure S3

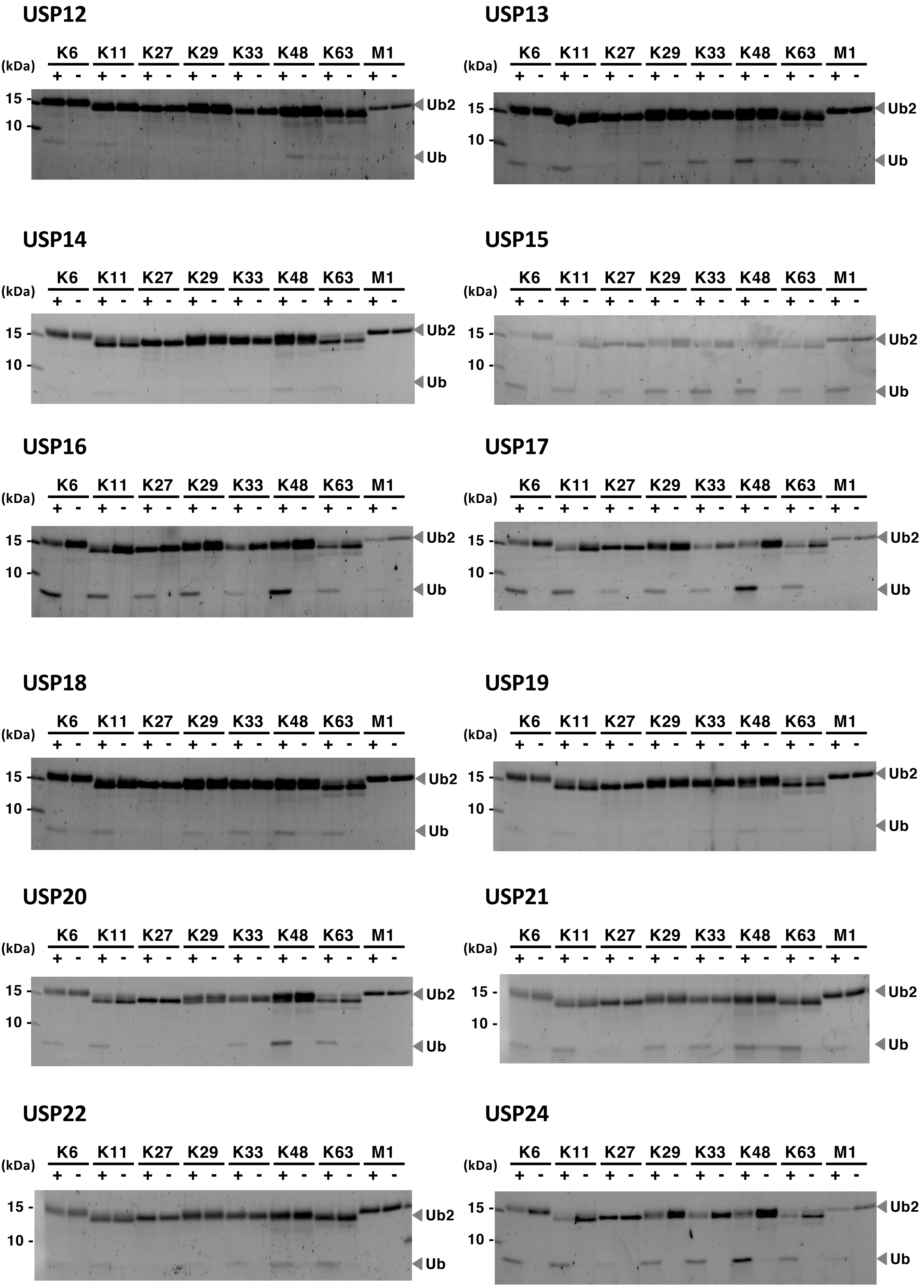
USP12
USP13
USP14
USP15
USP16
USP17
USP18
USP19
USP20
USP21
USP22
USP24
K6 K11 K27 K29 K33 K48 K63 M1
(kDa)
+ - + - + - + - + - + - + - + -
15
10
Ub2
Ub

Figure S3

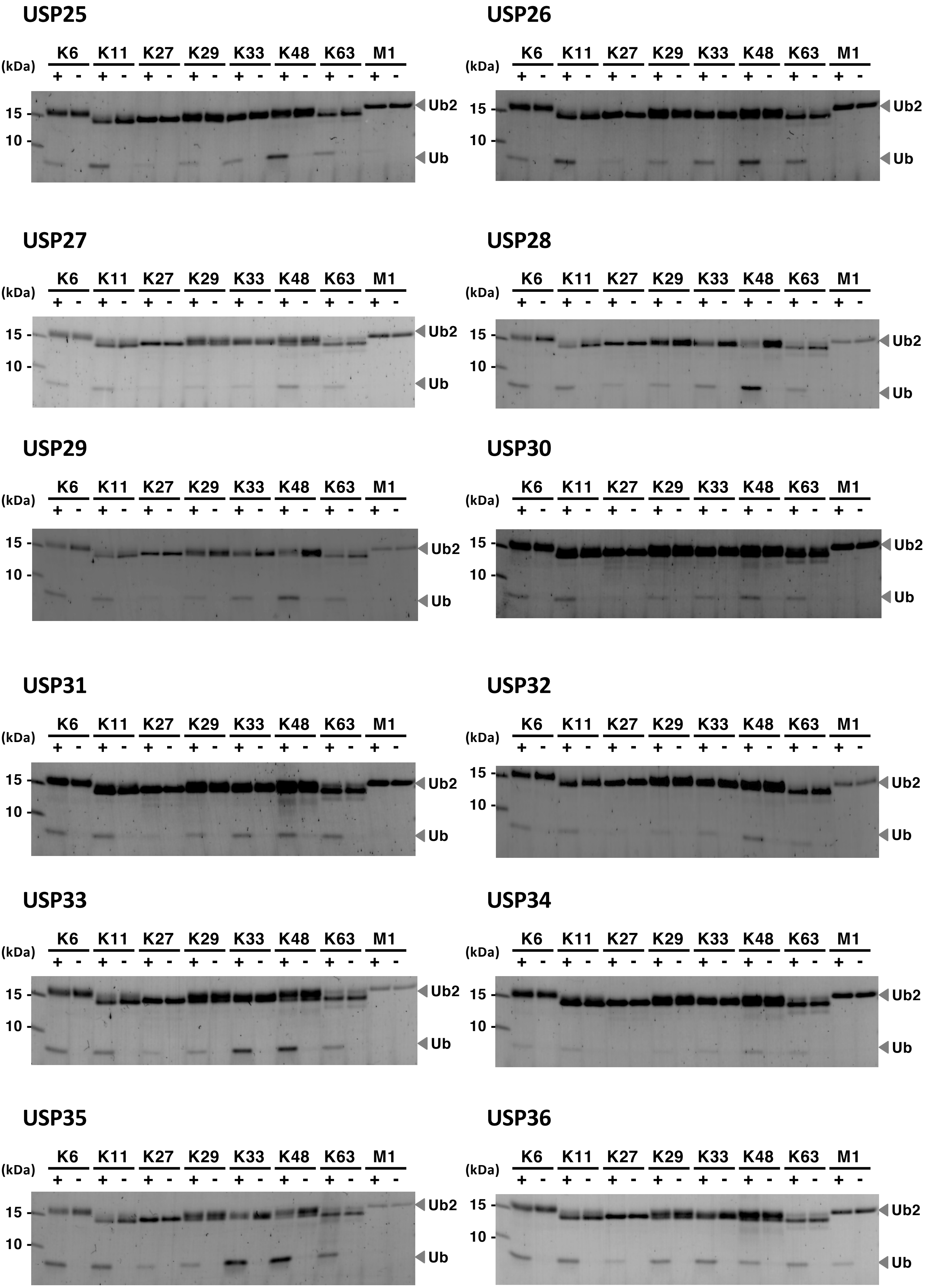
USP25
K6 K11 K27 K29 K33 K48 K63 M1
(kDa)
+ - + - + - + - + - + - + - + -
15
10
Ub2
Ub
USP26
K6 K11 K27 K29 K33 K48 K63 M1
(kDa)
+ - + - + - + - + - + - + - + -
15
10
Ub2
Ub
USP27
K6 K11 K27 K29 K33 K48 K63 M1
(kDa)
+ - + - + - + - + - + - + - + -
15
10
Ub2
Ub
USP28
K6 K11 K27 K29 K33 K48 K63 M1
(kDa)
+ - + - + - + - + - + - + - + -
15
10
Ub2
Ub
USP29
K6 K11 K27 K29 K33 K48 K63 M1
(kDa)
+ - + - + - + - + - + - + - + -
15
10
Ub2
Ub
USP30
K6 K11 K27 K29 K33 K48 K63 M1
(kDa)
+ - + - + - + - + - + - + - + -
15
10
Ub2
Ub
USP31
K6 K11 K27 K29 K33 K48 K63 M1
(kDa)
+ - + - + - + - + - + - + - + -
15
10
Ub2
Ub
USP32
K6 K11 K27 K29 K33 K48 K63 M1
(kDa)
+ - + - + - + - + - + - + - + -
15
10
Ub2
Ub
USP33
K6 K11 K27 K29 K33 K48 K63 M1
(kDa)
+ - + - + - + - + - + - + - + -
15
10
Ub2
Ub
USP34
K6 K11 K27 K29 K33 K48 K63 M1
(kDa)
+ - + - + - + - + - + - + - + -
15
10
Ub2
Ub
USP35
K6 K11 K27 K29 K33 K48 K63 M1
(kDa)
+ - + - + - + - + - + - + - + -
15
10
Ub2
Ub
USP36
K6 K11 K27 K29 K33 K48 K63 M1
(kDa)
+ - + - + - + - + - + - + - + -
15
10
Ub2
Ub

Figure S3

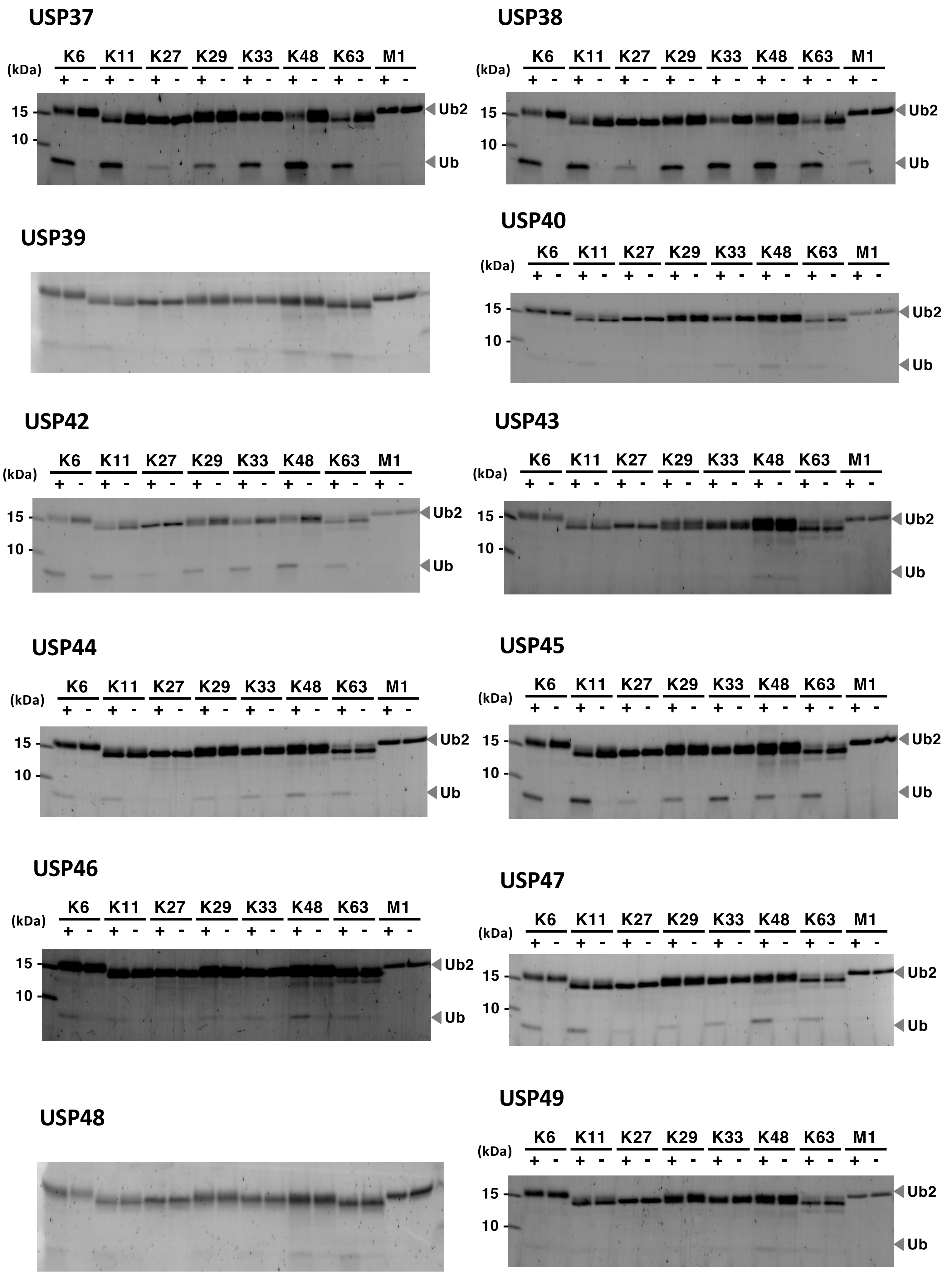
USP37
USP38
USP39
USP40
USP42
USP43
USP44
USP45
USP46
USP47
USP48
USP49
K6 K11 K27 K29 K33 K48 K63 M1
(kDa)
+ - + - + - + - + - + - + - + -
15
10
Ub2
Ub

Figure S3

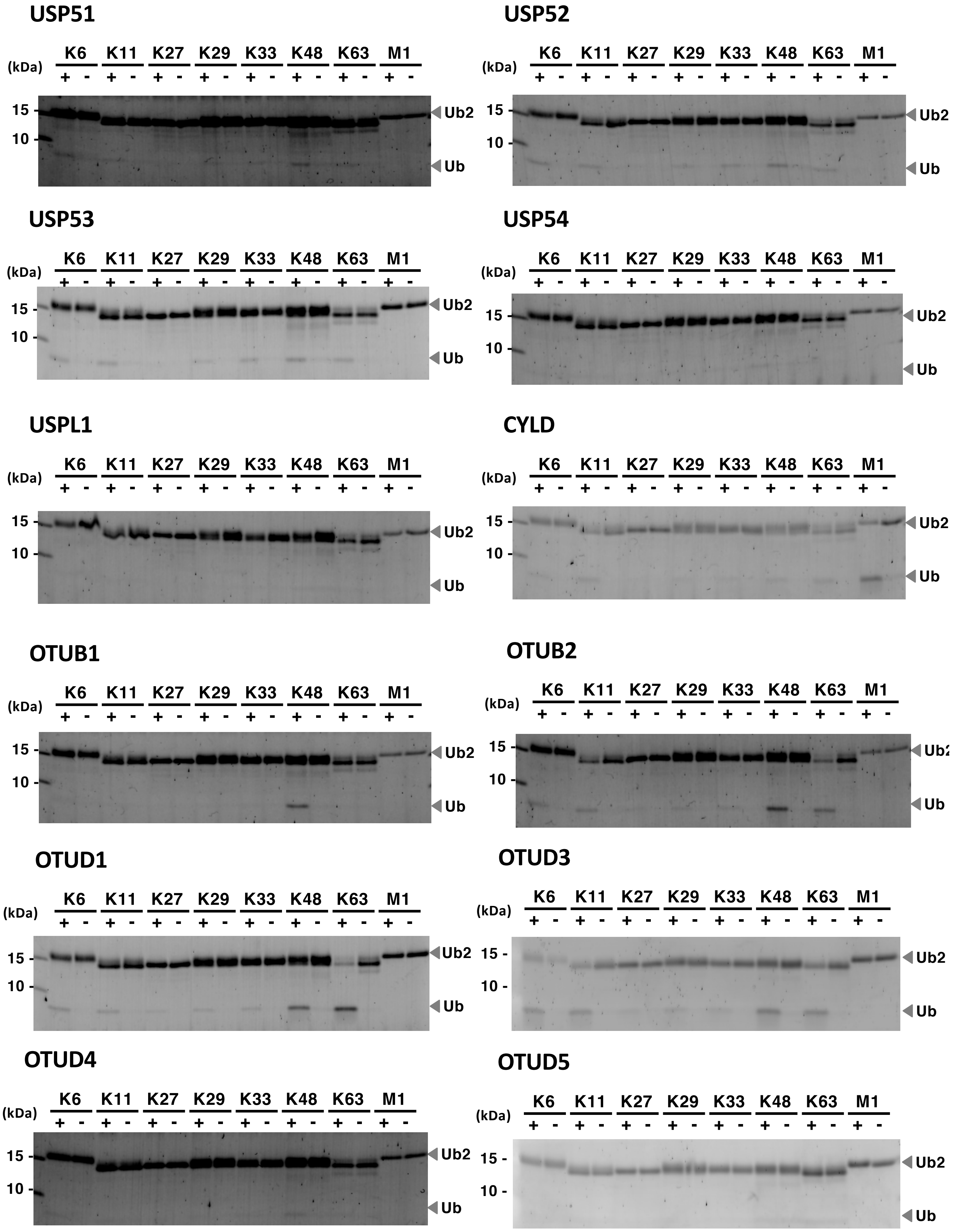
USP51
USP52
USP53
USP54
USPL1
CYLD
OTUB1
OTUB2
OTUD1
OTUD3
OTUD4
OTUD5
K6 K11 K27 K29 K33 K48 K63 M1
+ - + - + - + - + - + - + - + -
(kDa)
15
10
Ub2
Ub

Figure S3

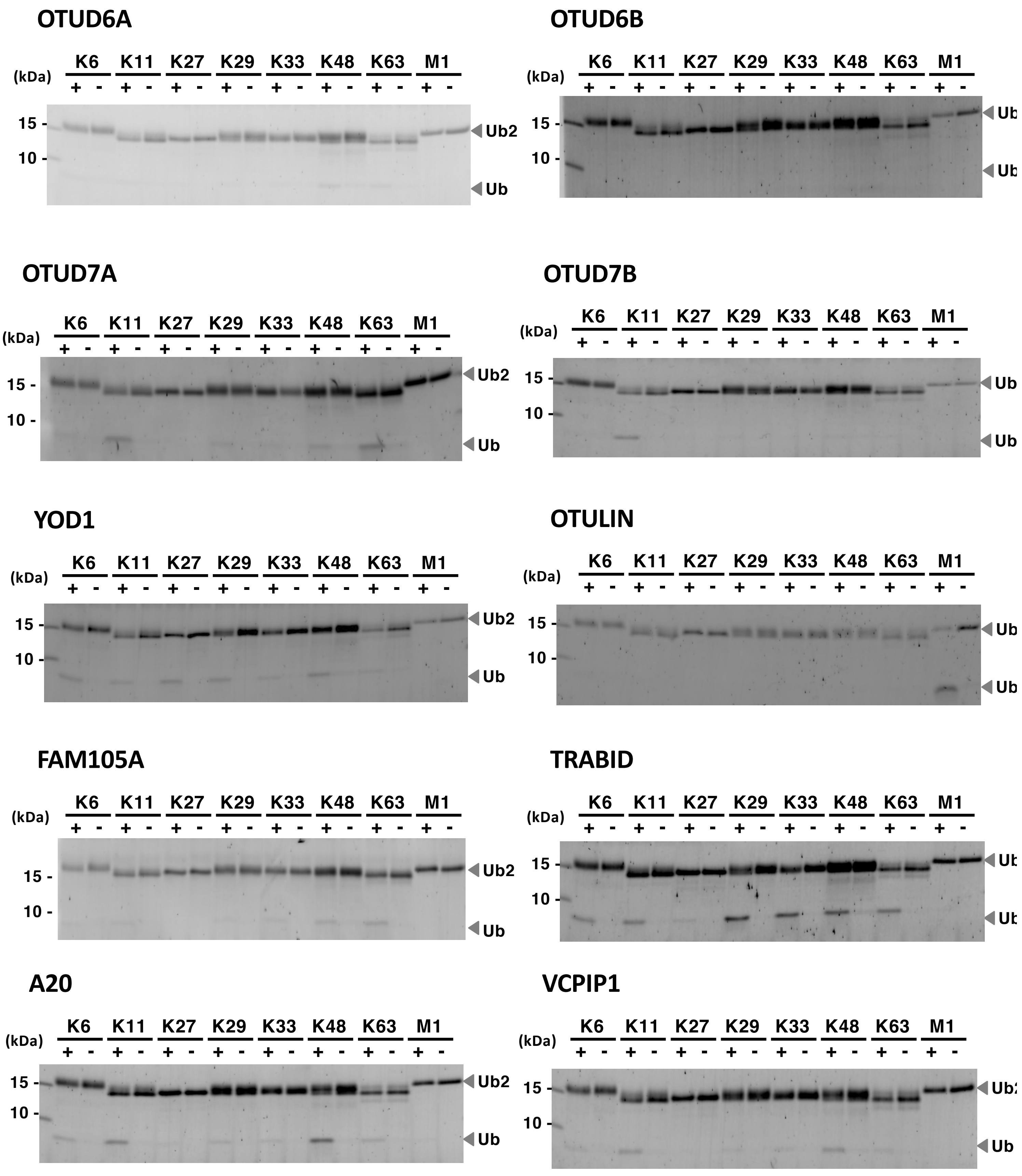

OTUD6A
OTUD6B
OTUD7A
OTUD7B
YOD1
OTULIN
FAM105A
TRABID
A20
VCPIP1
K6
K11
K27
K29
K33
K48
K63
M1
(kDa)
15
10
Ub2
Ub

Figure S3

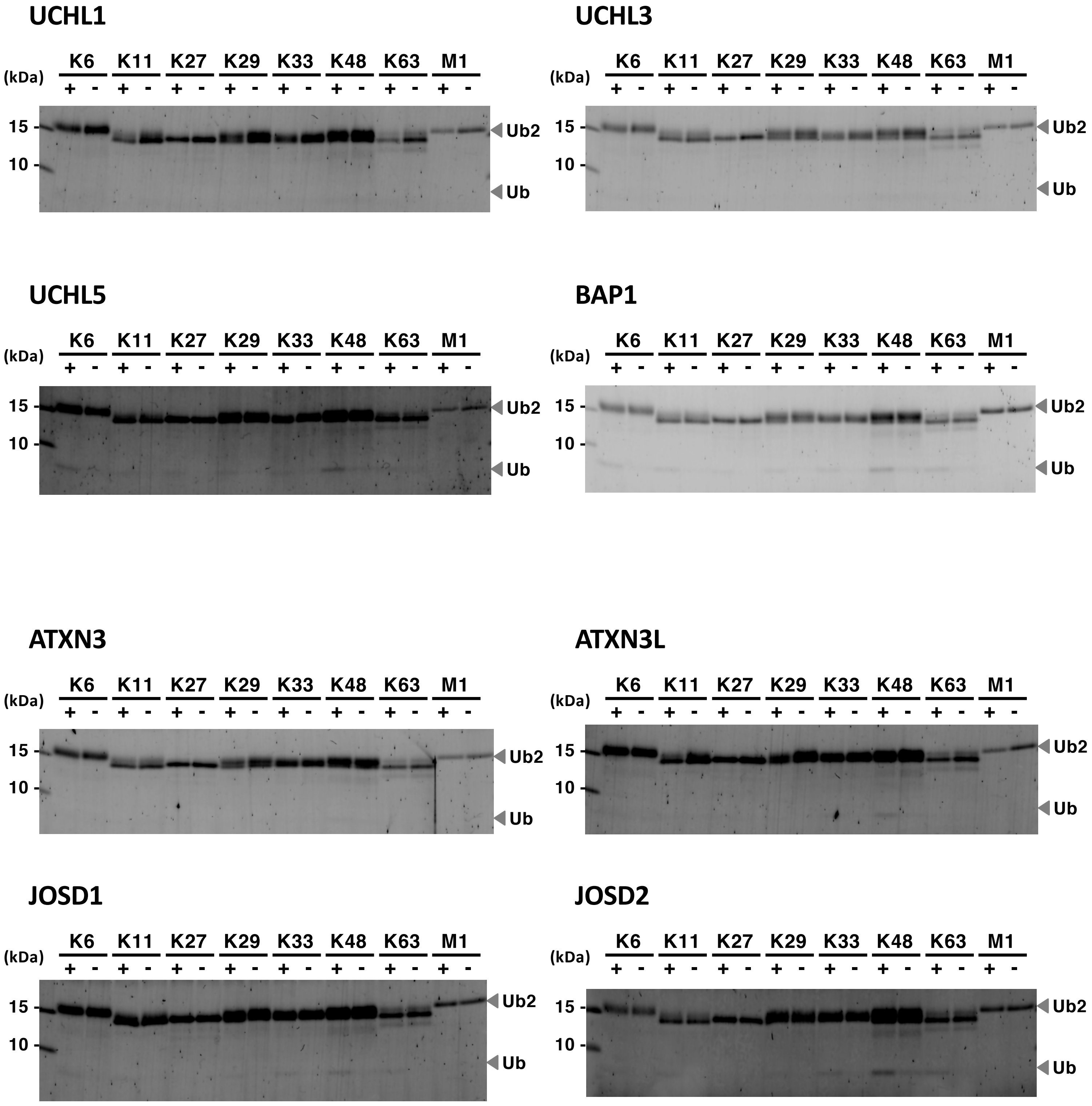
UCHL1
UCHL3
UCHL5
BAP1
ATXN3
ATXN3L
JOSD1
JOSD2
K6 K11 K27 K29 K33 K48 K63 M1
(kDa)
+ - + - + - + - + - + - + - + -
15
10
Ub2
Ub

Figure S3

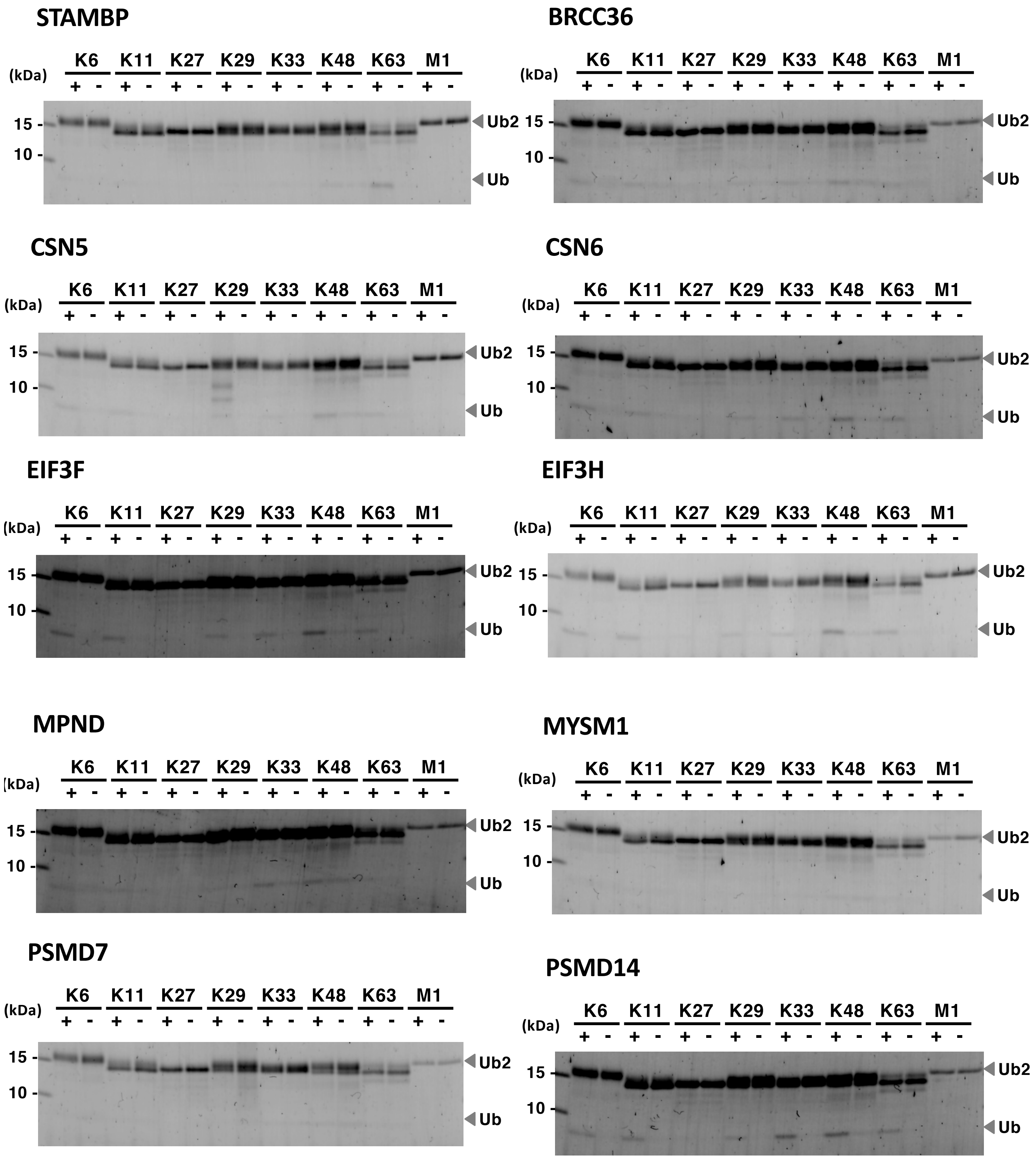

Figure S3. The result of in vitro DUB assay in Figure 3. All of the SYPRO Ruby staining gels after the DUB assay were shown.

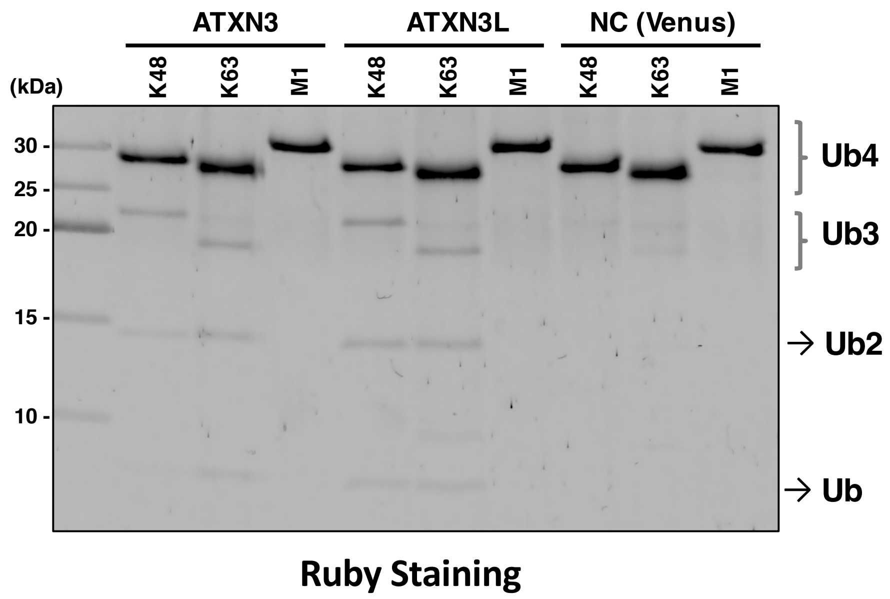

Figure S4. The result of DUB assay of ATXN3 and ATXN3L using tetraubiquitins as substrate. The in vitro DUB assay was carried out as same procedure as Figure 2, except for using K48-, K63-, and M1-tetraubiquitins were used.

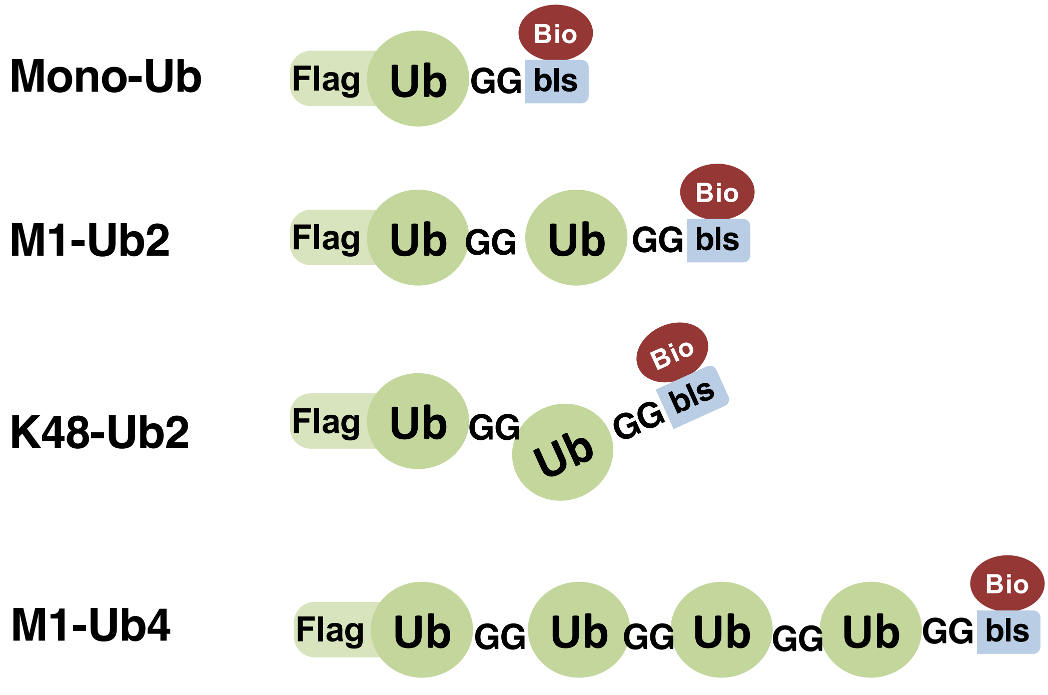

Figure S5. The schematic diagram of substrate ubiquitins for AlphaScreen-based DUB assay. Abbreviations are indicated as follows: Flag, FLAG-tag; Bio, biotin; bls, biotin ligase recognition sequence; Ub, ubiquitin.

Table S1. The list of all human DUBs. The other names, and predicted molecular weight and amino acid sequence of each DUB used in this study were indicated. DUBs that were not analyzed in this study are indicated in gray characters.

Table S2. The results of DUB assay using diubiquitins. The actual ratio of cleaved diubiquitins in Figure 3 are shown in column B to I. The concentration of each DUB used in the assay is determined by CBB staining (see detail in the materials and methods), and indicated in column J. N.D indicates the DUBs that we failed to determine the concentration, because the band of DUB staining with CBB was not obtained (*1) or was overlapped with the band of heavy chain of anti-AGIA antibody (*2). The comparison of the result of DUB assay between our result and previously published data is shown with following definition; The results between our assay and previous result are totally identical (Circle), partially agree (Triangle), and totally different (Cross mark).

Table S3. The results of the AlphaScreen-based evaluation of DUB inhibitors in Figure 4B. For individual DUB, substrates ubiquitin used and reaction time are shown in column C and D. The average of inhibition rate and its standard deviation from three independent assay were shown in E and H.
